# Trace Level Quantitation of Phenyltin Compounds Using HPTLC

**DOI:** 10.6028/jres.093.054

**Published:** 1988-06-01

**Authors:** K. K. Brown, P. Tomboulian, S. M. Walters

**Affiliations:** Department of Chemistry, Oakland University, Rochester, MI 48063; Pesticide and Industrial Chemical Research Center, U.S. Food and Drug Administration, Detroit, MI 48207; Department of Chemistry, Oakland University, Rochester, MI 48063; Pesticide and Industrial Chemical Research Center, U.S. Food and Drug Administration, Detroit, MI 48207

We sought to develop a rapid analytical technique for the determination of triphenyltin pesticide residues in food. HPTLC offers such an approach since it is rapid, selective, sensitive and has a high throughput [1]. The quantitative aspects of HPTLC have been documented and reviewed [2]. Conventional TLC has been used to separate butyltin compounds, subsequently detected using chemical oxidation and colorimetry of the pyrocatechol-complex [3]. TLC has also been used to speciate organotin compounds that were detected by anodic stripping voltammetry [4]. A study of the mammalian metabolism of organotins used normal and two-dimensional TLC, followed by photolysis and treatment with visualizing reagents (8-hydroxy-5-sulfonic acid, pyrocatechol violet, or dithizone) to identify the chromatographic components [5]. Another TLC method for organotins reported retention factor values for a series of tri-, di- and monosubstituted organotins; hematooxylin was used for visualization and the good resolving capacity of TLC was demonstrated [6]. HPTLC quantitation of butyltins in a wood extract matrix has been reported using post-development photolysis and colorimetric detection by complexation with pyrocatechol violet [7]. When complexed with inorganic tin and some organotins, morin produced a fluorescent complex; this principle forms the basis of this sensitive (DTL: 10^−7^ to 10^−9^ *M*) and selective analytical method [8].

The present study examines the use of HPTLC with in situ post-development derivatization using morin; fluorescence detection in HPTLC typically offers greater sensitivity and selectivity than other light absorption methods [9]. Fluorescence enhancement plate coatings are an important aspect of HPTLC quantitative analysis because they produce enhanced and stabilized fluorescence on HPTLC plates [16, 17].

Mixtures of tetraphenyltin (TTPT), triphenyltin chloride (TPTCl), diphenyltin dichloride (DPTCl_2_), and monophenyltin trichloride (MPTCl_3_) were resolved using high performance thin layer chromotography on silica gel with retention factor values of 0.80, 0.35, 0.20, 0.01, respectively. Inorganic tin impurities were strongly adsorbed and did not migrate from the origin. Diphenyltin dichloride, monophenyltin trichloride, and inorganic tin components reacted with morin to produce fluorescent complexes. Post-development exposure of the plate to ultraviolet light photodegraded the organic components which, after morin treatment, exhibited greater fluorescence than the organotins. This photolysis technique permitted the visualization of the otherwise nonfluorescent tetraphenyltin and weakly fluorescent triphenyltin spots.

The components were quantitated using scanning densitometry. The working range varied from a maximum of 222 nanograms to a minimum of 1 nanogram, depending on the particular component and the excitation wavelength chosen. Thirty standards, each containing five components, were spotted, developed, derivatized, and scanned at least three times to produce 480 pieces of data within 4 hours. Calibration curves showed an instrumental error of 1.5% relative standard deviation, and a spotter and intraplate variation of 9.0% relative standard deviation. The inherent multiplicity of high performance thin layer chromatography allows for multiple sampling and analysis, thereby yielding significantly increased precision and high sample throughput. The chromatography and detection of butyltins and cyclohexyltins were also examined.

The four chromatograms in figure 1 demonstrate the HPTLC of the compounds TTPT, TPTCl, DPTCl_2_, MPTCl_3_, and organic tin on silica gel using two different mobile phases, with and without the use of photolysis prior to morin derivatization and fluorescence detection. Chromatograms 1a and 1b used mobile phase A (5% acetic acid, 20% methylene chloride, 75% isooctane v/v) which completely resolves the organotins. However, MPTCl_3_ is not moved from the origin and if inorganic tin compounds are also present MPTCl_3_ is not resolved from them, therefore a stronger mobile phase in a separate development is required to resolve MPTCl_3_ from inorganic tin. The efficiency is excellent with no tailing or peak asymmetry. Photolysis increases the fluorescence of each compound, and is critical for the detection of TTPT, and TPTCl. Chromatograms 1c and 1d use a stronger mobile phase B (30% acetic acid, 70% chloroform v/v). The Rf values of TTPT, TPTCl, DPTCl_2_, and MPTCl_3_ were: 0.85, 0.55, 0.25, and 0.00 using mobile phase A; and 0.65, 0.56, 0.14, and 0.05 using mobile phase B. TPTCl, triphenyltin hydroxide (TPTOH), and triphenyltin acetate (TPTAc) all elute as TPTAc, as established using preparative TLC and FT-IR comparisons.

The calibration curves shown in figure 2 are for MTPCl_3_, DPTCl_2_, TPTCl, and TTPT with the analyte-morin complex excited at 313 nm. The error bars at each measured concentration level indicate the mean value, plus and minus one standard deviation (*N* = 6). The relative slope of each line correlates qualitatively to the percent of tin in each molecule; thus the detection limit is lowest for MPTCl_3_.

Figure 3 shows 15 chromatograms from authentic organotin reference compounds that were applied to a single 10-cm×10-cm HPTLC plate, and analyzed by the described method. The organotin samples and the corresponding peaks were: fenbutatin oxide (samples 1–3; peak A); triphenyltin oxide, TPTO (samples 4–6; peak B); monocyclohexyltin tribromide, MCyTBr_3_ (samples 7–9; peak C); dicyclohexyltin dibromide, DCyTBr_2_ (samples 7–9; peak D); tricyclohexyltin bromide, TCyTBr (samples 7–9; peak E); monobutyltin trichloride, MBTCl_3_ (samples 10–12; peak F); dibutyltin dichloride (samples 10–12; peak G); tributyltin chloride, TBTCl (samples 10–12; peak H); monophenyltin trichloride, MPTCl_3_ (samples 13–15; peak I); diphenyltin dichloride, DPTCl_2_ (samples 13–15; peak J); triphenyltin chloride (samples 13–15; peak K); tetraphenyltin, TTPT (samples 13–15; peak L). Each standard had an approximate mass of 10 ng except the MBTCl_3_, and MPTCl_3_ which were of uncertain quantity. The standards were eluted with mobile phase A to a 5-cm solvent front. The processed plate was scanned longitudinally with 15 4-cm passes to measure the fluorescence emitted at 515 nm (broad band pass filter) using 436 nm excitation. The results in figure 3 demonstrate that resolution of organotins between classes of compounds is excellent (i.e., R_4_Sn, R_3_SnX, R_2_SnX_2_, and RSnX_3_ where R=alkyl or aryl groups).

Currently work is being done to incorporate this determinative step to quantitate TPT residues in potatoes. Quantitation of TPT at 0.1 ppm has been accomplished. Potato extracts have been screened for TPT down to 0.01 ppm. This determinative method has also been used to measure tri-, di-, and monobutyltin chloride in water down to 0.01 ppm.

## Figures and Tables

**Figure 1:**
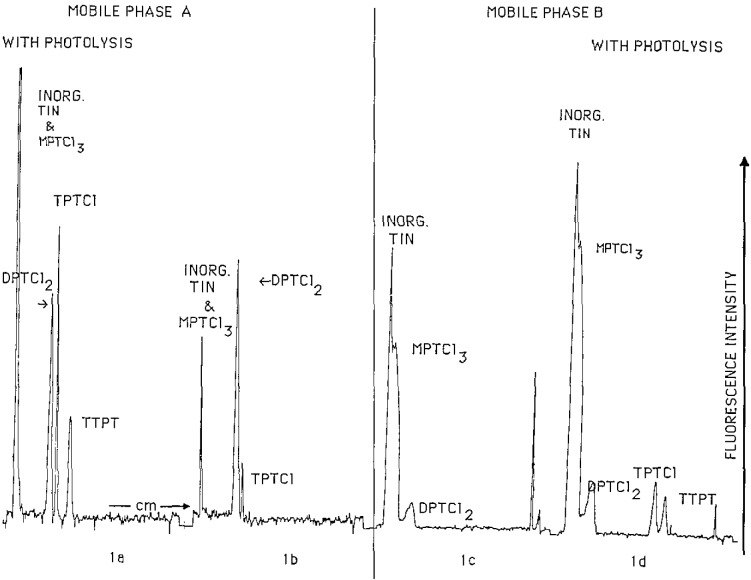
Four chromatograms separating inorganic tin, MPTCl_3_, DPTCl_2_, TPTCl, and TTPT. Chromatograms 1a and 1b used mobile phase A, and 1c and 1d used mobile phase B.

**Figure 2:**
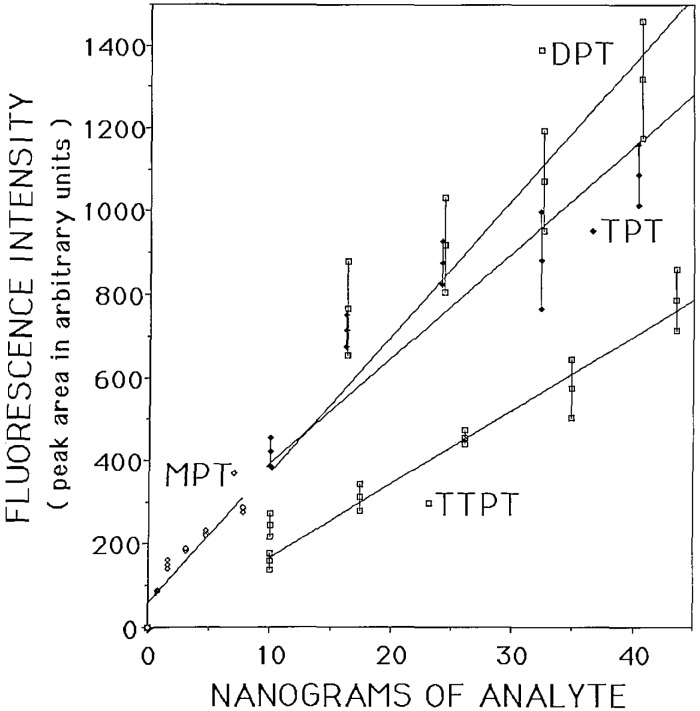
Fluorescence calibration curves for MPTCl_3_, DPTCl_2_, TPTCl, and TTPT excited at 313 nm. Error bars show ±SD.

**Figure 3:**
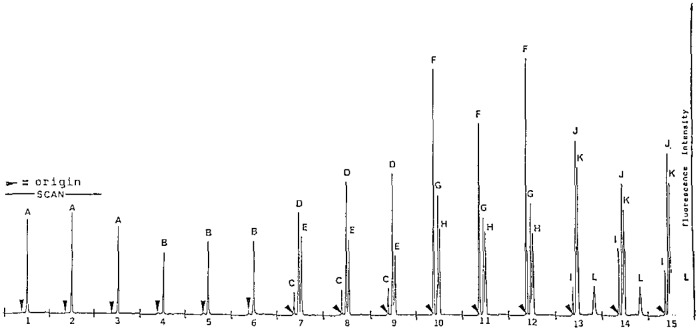
Fifteen chromatograms produced from 4 cm longitudinal densitometer scans across a 10 cm × 10 cm HPTLC plate with 15 organotin samples. Components: A, 6 ng fenbutatin oxide; B, 7 ng TPTO; C, MCyTBr_3_; D, 11 ng DCyTBr_2_; E, 11 ng TCyTBr; F, 11 ng MBTCl_3_; G, 11 ng DBTCl_2_; H, 13 ng TBTCl; 1, MPTCl_3_; J, 12 ng DPTCl_2_; K, 12 ng TPTCl; L, 13 ng TTPT.
